# A Biodegradable Microneedle Cuff for Comparison of Drug Effects through Perivascular Delivery to Balloon-Injured Arteries

**DOI:** 10.3390/polym9020056

**Published:** 2017-02-08

**Authors:** Dae-Hyun Kim, Eui Hwa Jang, Kang Ju Lee, Ji Yong Lee, Seung Hyun Park, Il Ho Seo, Kang Woog Lee, Seung Hyun Lee, WonHyoung Ryu, Young-Nam Youn

**Affiliations:** 1 Division of Cardiovascular Surgery, Severance Cardiovascular Hospital, Yonsei University College of Medicine, 50-1 Yonsei-ro, Sedaemun-gu, Seoul 03722, Korea; vet1982@hanmail.net (D.-H.K.); nurjih83@yuhs.ac (E.H.J.); sysebg@yuhs.ac (K.W.L.); henry75@yuhs.ac (S.H.L.); 2 School of Mechanical Engineering, Yonsei University, 50 Yonsei-ro, Sedaemun-gu, Seoul 03722, Korea; knjulee@gmail.com (K.J.L.); bamemo@naver.com (J.Y.L.); dwitong@naver.com (S.H.P.); kiayora@gmail.com (I.H.S.)

**Keywords:** anastomosis, drug delivery, microneedle, restenosis, peripheral vascular disease

## Abstract

Restenosis at a vascular anastomosis site is a major cause of graft failure and is difficult to prevent by conventional treatment. Perivascular drug delivery has advantages as drugs can be diffused to tunica media and subintima while minimizing the direct effect on endothelium. This in vivo study investigated the comparative effectiveness of paclitaxel, sirolimus, and sunitinib using a perivascular biodegradable microneedle cuff. A total of 31 New Zealand white rabbits were used. Rhodamine was used to visualize drug distribution (*n* = 3). Sirolimus- (*n* = 7), sunitinib- (*n* = 7), and paclitaxel-loaded (*n* = 7) microneedle cuffs were placed at balloon-injured abdominal aortae and compared to drug-free cuffs (*n* = 7). Basic histological structures were not affected by microneedle devices, and vascular wall thickness of the device-only group was similar to that of normal artery. Quantitative analysis revealed significantly decreased neointima formation in all drug-treated groups (*p* < 0.001). However, the tunica media layer of the paclitaxel-treated group was significantly thinner than that of other groups and also showed the highest apoptotic ratio (*p* < 0.001). Proliferating cell nuclear antigen (PCNA)-positive cells were significantly reduced in all drug-treated groups. Sirolimus or sunitinib appeared to be more appropriate for microneedle devices capable of slow drug release because vascular wall thickness was minimally affected.

## 1. Introduction

Application of biodegradable polymers for medical purposes—such as drug delivery, nanoparticle imaging technology, and soft tissue reconstruction—has been widely reported [1,2,3,4]. Recently, a lot of polymeric devices such as films/wraps, meshes, rings, or micro/nano particles were also introduced for drug delivery to various cardiovascular lesions [5,6,7,8,9,10].

Cardiovascular disease is a major cause of death worldwide [11]. In particular, obstructive diseases of small-sized vessels like myocardial infarction have the highest mortality rates among various cardiovascular diseases [11]. Although continuous progress has been made, successful implementation of non-surgical treatments such as balloon angioplasty and endovascular stenting remains challenging due to restenosis derived from neointimal hyperplasia [12,13].

When vascular endothelium is damaged by any cause, smooth muscle cells (SMCs) migrate to an intima layer of the vessel and excessively proliferate, leading to neointimal hyperplasia [14]. Therefore, various attempts have been made to locally deliver anti-proliferative agents, such as paclitaxel and sirolimus, via the use of drug-eluting stent (DES) or drug-eluting balloons [15,16,17,18]. Although promising-inhibitory effects on neointimal hyperplasia have been reported in various preclinical and clinical studies, these methods are limited in enabling sustained, controlled drug delivery to the tunica media layer, or as effectively as desired [19,20]. In addition, loss of vascular patency due to late stent thrombosis or restenosis was reported in up to 20% of patients in long-term follow-up studies on DES [12,13].

When restenosis and intimal hyperplasia cannot be limited by intraluminal therapy, arterial replacement or bypass surgery is currently accepted as a general surgical treatment method [11]. However, the occurrence rate of restenosis due to neointimal hyperplasia at vascular anastomosis sites is also relatively high in small-sized vessels less than 6 mm in diameter [11]. In several studies, extraluminal drug delivery using a perivascular biodegradable material was reported to reduce the recurrent rate [12,21]. Such an approach is advantageous as drugs can be diffused to not only the tunica adventitia but also tunica media and subintima while minimizing the direct effect on endothelium [12]. 

To explore this advantage further, we developed a perivascular cuff containing an array of microneedles (MN) that has been shown to drastically increase drug delivery efficiency compared with devices without MN [20,22]. In addition, the device itself did not affect normal vascular structures and demonstrated a capability to effectively inhibit neointima formation by delivering paclitaxel to the vascular tissue layer [20]. However, thinning of the tunica media layer was observed in paclitaxel-loading groups on histopathological examination. 

In the present study, we thus aim to compare the effects other anti-proliferating drugs with paclitaxel on inhibition of neointima formation as well as thinning of tunica media when delivered through a MN cuff. Specifically, sirolimus is widely used in DES and has been shown to inhibit neointimal hyperplasia effectively through cytostatic mechanisms while sunitinib malate salt (sunitinib) was recently reported to show an inhibitory effect on neointimal hyperplasia. Therefore, these two drugs were compared with paclitaxel.

## 2. Methods

### 2.1. MN Device Fabrication

**Figure 1:**
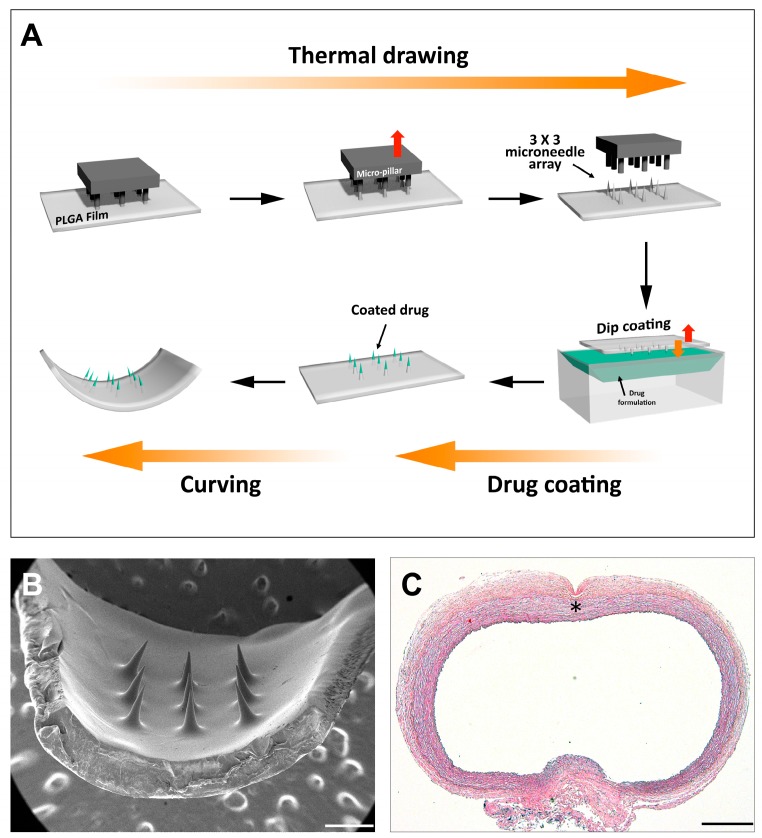
(**A**) A schematic diagram of microneedle cuff fabrication; (**B**) A SEM image of microneedle cuff (the scale bar indicates 500 μm); (**C**) A Microscopic image of an abdominal aorta with the microneedle insertion. The asterisk indicates the insertion mark by the microneedle cuff device (hematoxylin and eosin staining; the scale bar indicates 500 μm).

The MN cuff was fabricated through the following steps, as previously reported: MN array fabrication by thermal drawing, dip coating of drug onto MN end tips, and post-annealing for cuff shape form (Figure 1) [20,22]. Briefly, a biodegradable polymer film of 100 µm thickness was fabricated with poly(lactic-*co*-glycolic acid) (PLGA) 90/10 (*M*_w_ = 268,000, Samyang Biopharmaceuticals, Gyeonggi-do, Korea). Subsequently, a 3 × 3 array of micro-pillars attached to a heating cartridge was lowered toward the PLGA film on a hot chuck. After making contact between the micro-pillars and PLGA film, the micro-pillar array was lifted up with an automatic micro-stage and this motion resulted in thermal drawing of the heated PLGA. Detailed shapes of the MNs were adjusted by carefully modulating pillar and film temperature, drawing speeds, drawing steps, and drawing distance for each step.

**Figure 2:**
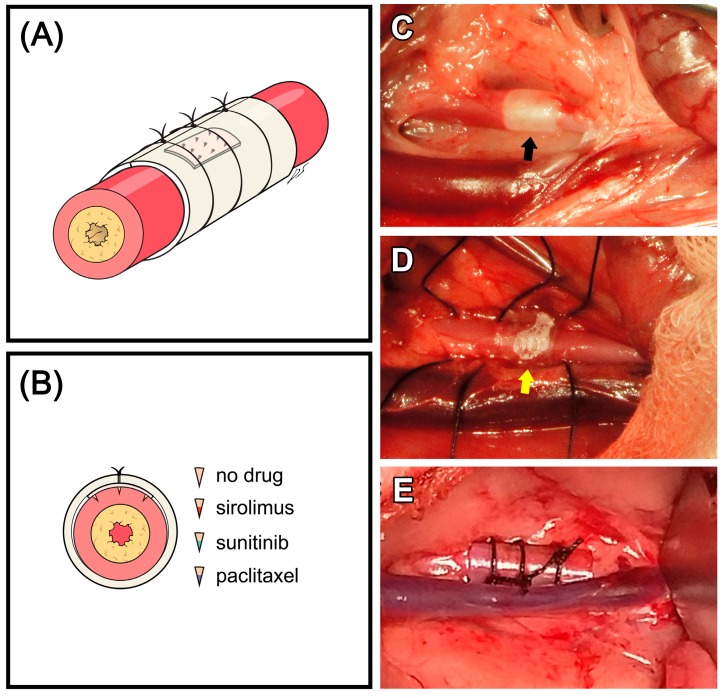
(**A**) A schematic view of microneedle device installation on an artery post induction of neointimal hyperplasia; (**B**) Cross-sectional view of the device installed on the artery and scheme of small box); each experimental group; (**C**) Balloon injury before MN device installation (black arrow: saline-inflated balloon); (**D**) MN device (yellow arrow) over the abdominal aorta; (**E**) Tightly-fixed MN device with Tygon tube and silks.

Paclitaxel, sirolimus, and sunitinib (P9600, R5000 and S-8803, LC Laboratories, Woburn, MA, USA) were used as anti-proliferative drugs in this study (Figure 2A,B). The drug formulations for sustained perivascular drug delivery were mixed at a 3:1:0.33 weight ratio of DMSO, PLGA50/50 (PDLG5010, PURAC, Gorinchem, The Netherlands), and anti-proliferative drug. As previously reported, 1.2 µg of each drug was loaded into MN end tips by dip coating with the drug formulations [23]. Then MN devices were annealed in a stainless cylinder 3 mm in diameter to form a cuff shape.

### 2.2. Animal Experiment

A total of 31 healthy male New Zealand white rabbits weighing 3.3 ± 0.2 kg (range: 2.9–3.6 kg) were used in the present study. Animal care and experimental procedures were conducted in accordance with the guidelines approved by the Institutional Animal Care and Use Committee (IACUC) at Yonsei University Health System, Seoul, Korea (IACUC approval No. 2015-0020, 2015).

The following experimental groups were compared in terms of the delivery effectiveness and the anti-neointimal formation effect of each drug after arterial balloon injury: rhodamine group (*n* = 3), fresh artery + rhodamine B-loaded MN device; I + D group (*n* = 7), balloon injury + MN device only; I + Sir group (*n* = 7), balloon injury + sirolimus-loaded MN device; I + Sun group (*n* = 7), balloon injury + sunitinib-loaded MN device; and I + Ptx group (*n* = 7), balloon injury + paclitaxel-loaded MN device. Samples of normal aorta were obtained from three New Zealand white rabbits used in tracheal transplantation-associated research, which was unrelated to vascular pathology (IACUC approval No. 2014-0172-2, 2014).

Each animal was intramuscularly injected with 5 mg/kg of xylazine and 15 mg/kg of Zoletil^®^ every 15 min as a premedication. After intubation with a 3.0 or 3.5 mm endotracheal tube, 1.5%–2.0% isoflurane was used to maintain inhalation anesthesia. All the animals received crystalloid solution (10 mL/kg/h) throughout the surgical procedure. 

After each animal was anesthetized, an abdominal midline incision was performed. The bowel was retracted to expose the abdominal aorta, and blunt dissection was performed to separate surrounding connective tissue. Then the right inguinal area was incised to expose the right femoral artery. Heparin (100 U/kg) was administered intravenously. A 3-Fr Fogarty embolectomy catheter was inserted through the right femoral artery and positioned in the abdominal aorta. The abdominal aorta was de-endothelialized with three passes of a balloon inflated with 0.05 ml saline (Figure 2C). The MN device was then positioned and fixed with a Tygon^®^ tube and three 4–0 silks (Figure 2D,E). After removing the balloon catheter, the right femoral artery was ligated, and the abdominal cavity, subcutaneous tissue, and skin were closed with general procedures. As analgesic, 1 mg/kg of ketolorac tromethamine was administered intramuscularly, two times per day for one week. For antibiotics, 5 mg/kg of enrofloxacin was administered subcutaneously, two times per day for one week. Aspirin (10 mg/kg) was administered orally once per day for one week.

### 2.3. Fluorescent Microscopic Analysis

To visualize post-delivery drug distribution within vascular tissue, a fluorescent model drug, rhodamine B, was used for a two-week in vivo study. For sampling, the animal was induced into anesthesia using the same method as described above, and 100 U/kg of heparin was administered intravenously. Vascular clamps were applied at the cranial and caudal portions of the artery around the device location. After collecting samples by en-bloc resection, animals were euthanized by bolus injection of high-dose KCl.

The collected samples were trimmed to appropriate sizes after removing the MN devices. They were then put into molds, embedded into optimal cutting temperature (OCT) compound, frozen in a −80 °C deep freezer, and finally cryosectioned to 5-μm thickness. Fluorescent images were taken using a computer-assisted image analysis program (DP Controller, Olympus), with exposure time maintained as 0.6 seconds at ISO 100. Under the same conditions, fluorescent images of normal vessels were taken as a reference control.

### 2.4. Histopathological Examinations

For histopathological analysis, animals were euthanized in the same method described above, and samples were collected. Each sample was processed using standard methods and embedded in paraffin, then sectioned to 4-μm thickness and stained. 

Hematoxylin (Sigma-Aldrich, St. Louis, MO, USA) and eosin (BBC Biochemical, Mt Vernon, WA, USA) staining was used to identify basic pathological changes such as vessel wall thickness, narrowing of vessel lumen, and neointimal formation. Movat’s pentachrome staining (Movat pentachrome stain kit, Empire Genomics, Buffalo, NY, USA) was used to identify detailed vessel components. 

For immunohistochemistry, the sections were deparaffinized using standard protocols, and antigen retrieval was performed with proteinase K. Then, sections were incubated for 10 min in 3% hydrogen peroxide (Duksan Hydrogen peroxide 3059, Gyeonggi-do, Korea) to inactivate endogenous peroxidase, and blocked by incubating for 1 h in 5% bovine serum albumin. Subsequently, the sections were labelled with the mouse anti-PCNA antibody (AbD Serotec, MCA1558). The antibody-labelled sections were then incubated with Dako EnVision+ System-HRP-labelled polymer antimouse kit solution (Dako4001, Denmark). DAP (K-3468, Dako, Glostrup, Denmark) staining was performed for 3 min at room temperature for tissue visualization. Sections were then counterstained with hematoxylin. Microscopic assessment was conducted by an independent pathologist (from a different institution) who was blinded to the treatment allocation.

Quantitative analysis was performed using Image J software (National Institutes of Health, Bethesda, MD, USA). A layer inside the internal elastic lamina was measured as the area of the neointima, and another layer between the internal and external elastic lamina was measured as the area of the tunica media. The extent of stenosis was determined as follows: % neointimal formation = neointimal area × 100/(neointimal + luminal area). The average tunica media thickness from eight sites in each vessel was compared.

### 2.5. TUNEL (Terminal Deoxynucleotidyl Transferase dUTP Nick end Labeling) Assay 

To compare the percentage of apoptotic vascular SMCs in the tunica media, TUNEL immunohistochemical staining (In Situ Cell Death Detection kit, Roche, Mannheim, Germany) was performed, and nuclear fast red staining was performed as counterstaining. Microscopic images at 400× magnification from eight sites of each vessel were used for apoptotic cell counting. Apoptotic ratio (%) was calculated as the ratio of apoptotic nuclei to total cell nuclei.

### 2.6. Statistical Analysis

All data are expressed as mean ± standard deviation. Statistical analyses were performed using GraphPad Prism 5.0 software (GraphPad Software, Inc., San Diego, CA, USA). Normal data distribution was determined using Shapiro-Wilk test. One-way ANOVA and post hoc Bonferroni tests were used to compare mean tunica media thickness, TUNEL-positive cells, and PCNA-positive cells between groups. Kruskal-Wallis test and Mann Whitney test were applied for testing overall/pair-wise group mean differences in other quantitative analysis results between groups. A *p*-value < 0.05 was considered statistically significant.

## 3. Results

### 3.1. Drug Distribution in Vascular Tissue by MN Device

**Figure 3:**
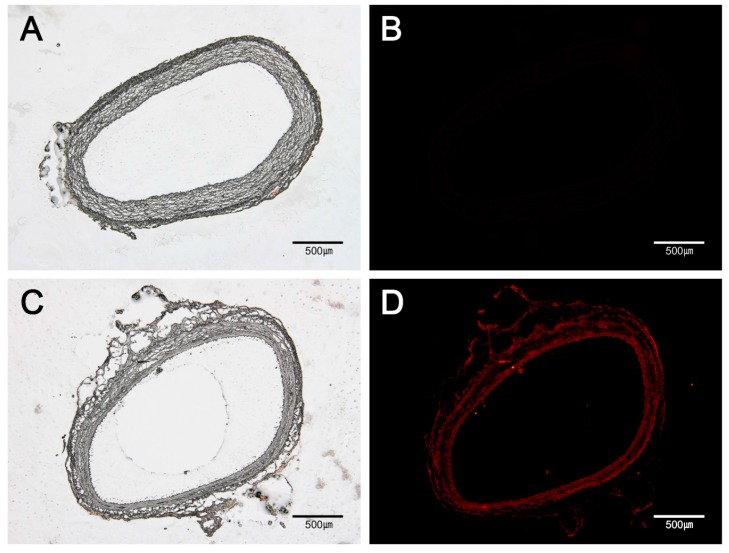
Fluorescent images of drug distribution. Compared to that in normal abdominal aorta (**B**), well-distributed rhodamine B delivered by the MN device is shown as red color in vascular tissue (**D**). (**A**) A light microscopic image of normal abdominal aorta; (**B**) A fluorescent image of normal abdominal aorta; (**C**) A light microscopic image of rhodamine B-delivered abdominal aorta; (**D**) A fluorescent image of rhodamine B-delivered abdominal aorta. The bar indicates 500 μm in all micrographs.

Fluorescent imaging of tissue sections confirmed rhodamine B was distributed almost in the entire vascular tissue. Fluorescence signal of rhodamine was detected in not only tunica adventitia, but also tunica media layer and subintima (Figure 3).

### 3.2. Quantitative Analysis

**Figure 4:**
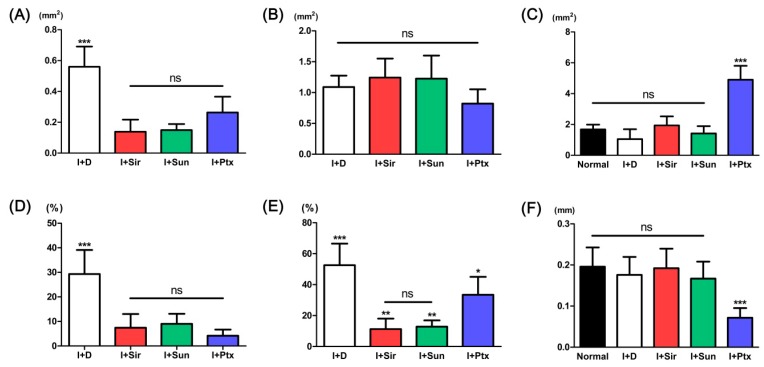
Quantitative analysis of neointimal area (**A**); tunica media area (**B**); luminal area (**C**); neointimal formation (**D**); ratios of neointima and tunica media area (**E**); and tunica media layer thickness (**F**). * *p* < 0.05 when compared with I + D group, ** *p* < 0.01 when compared with I + Ptx group, *** *p* < 0.001 when compared with other groups, ns = no significant difference.

The quantitative analysis results are presented in Table 1 and Figure 4. The neointimal area was significantly decreased in all drug treated groups (Figure 4A). The measured area of the tunica media showed no statistical difference between groups (Figure 4B). The measured area of the vessel lumen of sirolimus- (I + Sir) or sunitinib- (I + Sun) treated group did not statistically differ from the area of normal vessel or drug-free (I + D) group (Figure 4C). However, the paclitaxel- (I + Ptx) treated group showed significant dilatation compared to both the normal vessel and the groups treated with other anti-proliferative drugs. Neointimal formation (%) was significantly decreased in all drug-loaded groups with no statistical significance between drug-treated groups (Figure 4D). The ratio of tunica media area to neointimal area was also significantly decreased in all drug-loaded groups (Figure 4E). The measured thickness of the tunica media layer of normal vessel, I + D group, I + Sir group, and I + Sun group showed no statistical difference between the groups (Figure 4F). However, tunica media thickness in the I + Ptx group was significantly thinner than other groups.

### 3.3. Histopathological Findings

**Figure 5:**
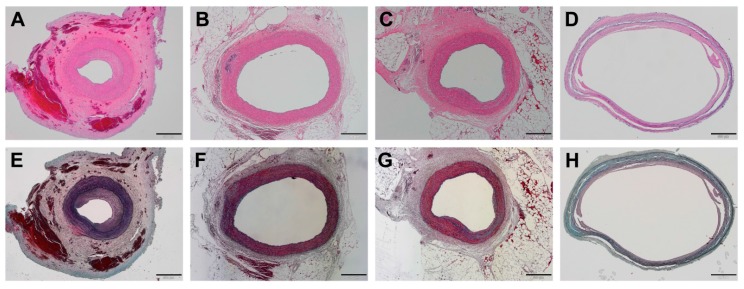
Low magnification images at four weeks after MN device installation. Note that distinctively thin tunica media layers with vascular dilatation were observed in the paclitaxel-treated group (**D**,**H**). (**A**,**E**) I + D group (animal No. 4); (**B**,**F**) I + Sir group (animal No. 11); (**C**,**G**) I + Sun group (animal No. 12); (**D**,**H**) I + Ptx group (animal No. 17). ((**A**–**D**): hematoxylin and eosin staining, (**E**–**H**): Movat’s pentachrome staining; the bar indicates 500 μm in all micrographs).

**Figure 6:**
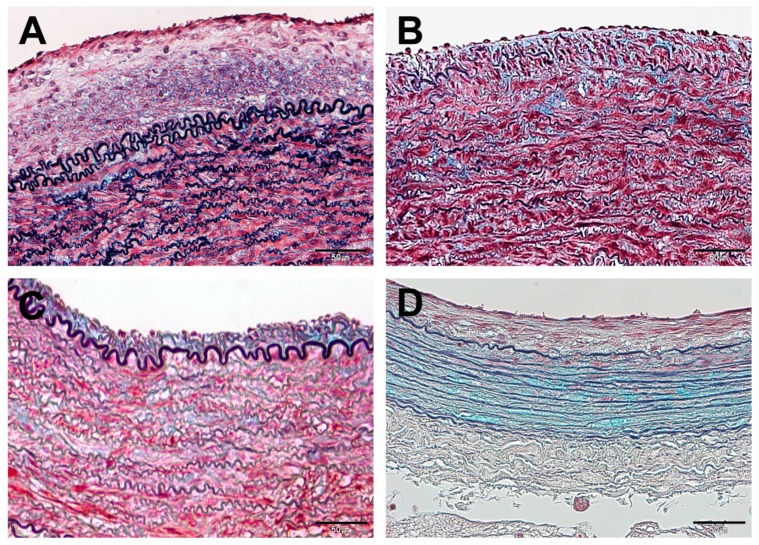
High magnification images at four weeks after MN device installation (Movat’s pentachrome staining; the bar indicates 50 μm in all micrographs). All drug-treated groups (**B**–**D**) displayed limited formation of neointima compared to the device-only group (**A**). The smooth muscle cells were rarely seen in the paclitaxel-treated group (**D**). (**A**) I + D group (animal No. 7); (**B**) I + Sir group (animal No. 8); (**C**) I + Sun group (animal No. 13); (**D**) I + Ptx group (animal No. 16).

In the I + D group, excessive formation of neointima was apparent in all animals (Figure 5A,E). Under high magnification, the neointima showed typical vascular scar tissue comprised of abundant SMCs of reddish-purple color and collagenous matrix of blue color on Movat’s pentachrome staining (Figure 6A). In addition, some breakdown of the elastic lamina was observed at the tunica media layer (Figure 6A).

The I + Sir group (Figure 5B,F) and I + Sun group (Figure 5C,G) showed similar histological features. They displayed limited formation of neointima compared to the I + D group, and neointima consisting of few SMCs and bluish collagen matrix was revealed with pentachrome stain (Figure 6B,C). Abundant SMCs and elastic lamina were also observed at the tunica media layer in both groups (Figure 6B,C).

In the I + Ptx group, a lesser degree of neointima formation was observed compared to the I + D group (Figure 5D,H). However, the tunica media layer was distinctively thinner (Figure 6D). It appeared mostly blue in color on pentachrome staining, because the cellularity of the tunica media layer was significantly lower and thus the staining was predominantly of the collagen matrix (Figure 6D).

**Figure 7:**
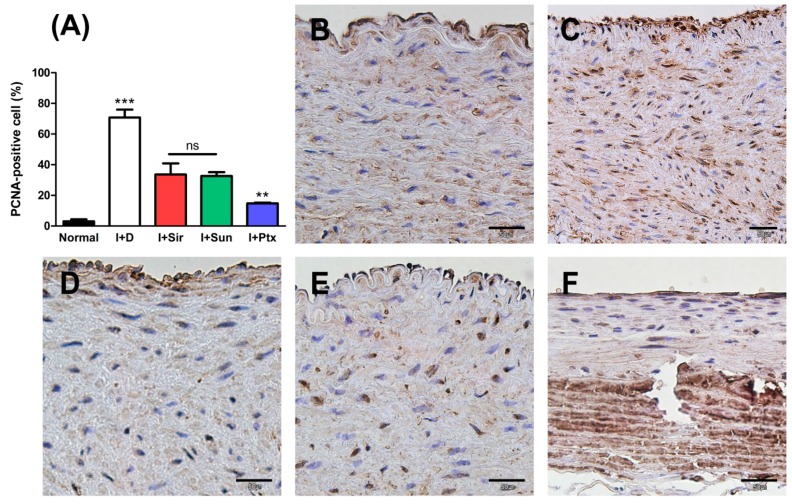
Anti-PCNA immunohistochemical results at four weeks after MN device installation. PCNA-positive cells were significantly reduced in all drug-treated groups. (**A**) A graph of the ratios of PCNA-positive cells to all nucleated cells. ** *p* < 0.01 when compared with I + Sir group and I + Sun group. *** *p* < 0.001 when compared with other groups, ns = no significant difference; (**B**) normal abdominal aorta; (**C**) I + D group (animal No. 5); (**D**) I + Sir group (animal No. 9); (**E**) I + Sun group (animal No. 13); (**F**) I + Ptx group (animal No. 16). The bar indicates 50 μm in all micrographs.

When compared to normal vessel, the ratio of PCNA-positive cells in all MN device groups was statistically significantly higher (*p* < 0.001) (Figure 7). However, the ratio of PCNA-positive cells of all drug-treated groups were significantly lower than those of the I + D group (*p* < 0.001). In addition, the I + Sun group and I + Sir group did not statistically differ, but I + Ptx group was significantly lower than both groups (*p* < 0.01).

### 3.4. Apoptosis of Vascular Smooth Muscle Cells

**Figure 8:**
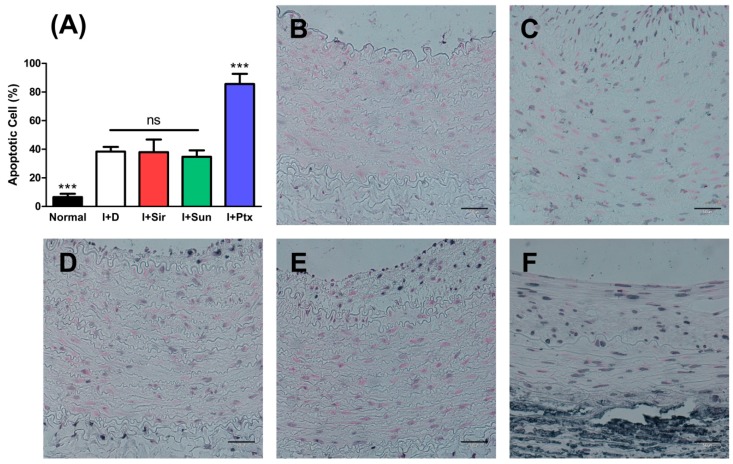
TUNEL staining results at four weeks after MN device installation. The paclitaxel-treated group (**F**) showed the highest apoptotic ratio. (**A**) A graph of apoptotic ratio (%). *** *p* < 0.001 when compared with other groups, ns = no significant difference; (**B**) normal abdominal aorta; (**C**) I + D group (animal No. 7); (**D**) I + Sir group (animal No. 10); (**E**) I + Sun group (animal No. 15); (**F**) I + Ptx group (animal No. 17). The bar indicates 50 μm in all micrographs.

When compared to a normal vessel, the apoptotic ratio in all MN device groups was statistically significantly higher (*p* < 0.001) (Figure 8). About 85% SMCs in the I + Ptx group displayed apoptosis, and the apoptotic nuclei ratio was significantly higher (*p* < 0.001) compared to all other groups. The I + Sir group, I + Sun group, and I+D group did not statistically differ. 

## 4. Discussion

In this study, the effectiveness of an MN-based drug delivery system was demonstrated by showing its inhibitory effect on neointimal formation in balloon-injured arteries using paclitaxel-, sirolimus-, and sunitinib-loaded MN device. 

Perivascular wraps fabricated from a biodegradable polymer such as poly(ε-caprolactone) (PCL) or PLGA have been introduced as a form of extraluminal drug-delivery device [24,25]. As mentioned above, one of the advantages of an extraluminal device is that the drug not only remains at the tunica externa layer but also gradually diffuses to the tunica media and subintima [12]. However, despite several successful reports in animal studies, application to human clinical trials has been relatively slow thus far, and related reports are lacking [12]. In addition, due to the possibility of drug loss to the non-contacted part of the vessel, delivering the desired amount of drug to the blood vessel wall remains a challenge [20]. To increase the efficacy of drug delivery, an intraluminal device employing a needle for the direct injection of drug into the adventitia has also been introduced [26]. However, this method is inappropriate for sustained delivery and still has the disadvantage of possible damage to vascular endothelium [12]. The perivascular MN device used in this study enables sustained delivery for a few weeks to months, with local drug delivery directly to the vascular tissue and high delivery efficiency.

We previously performed an animal study of the MN device using paclitaxel as a model drug [20]. Although neointimal formation was effectively inhibited, more than 90% of SMCs were identified as TUNEL-positive cells in an additional apoptosis study, as was similarly reproduced in the present study. Unlike sirolimus, paclitaxel is known to induce apoptosis of SMCs in S phase and G2/M phase in injured arteries [27]. The higher percentage of apoptotic cells observed with paclitaxel than with the other drugs in the present study is thought to be due to this mechanism. The very high ratio of TUNEL-positive cells is ascribed to the sustained drug release from the device. In addition, apoptosis induced by paclitaxel could have occurred in other cells related to the vascular remodeling such as fibroblasts in the tunica adventitia. Therefore, it is likely that an abnormal remodeling process after balloon injury contributed to the thinning of the arterial wall observed in this study.

Sirolimus is also a widely-used drug for the prevention of restenosis. A number of studies have reported different effects on the vessel due to different mechanisms of action between sirolimus and paclitaxel [27,28,29,30]. Pires et al. reported that high-dose paclitaxel increased the number of apoptotic cells and decreased SMCs and collagen content of the tunica media, while the arteries were not significantly affected by sirolimus treatment [29]. In addition, according to the study of Parry et al., the perivascular application of both drugs to the rat carotid artery injury model led to effective inhibition of neointimal hyperplasia; however, paclitaxel induced apoptotic cell death while sirolimus mainly inhibited neointimal formation by a cytostatic mechanism [27]. These results are consistent with our observation of a lower ratio of apoptotic cells in the sirolimus group than in the paclitaxel group.

There is some evidence that platelet-derived growth factor (PDGF) is associated with SMCs migration and proliferation during neointimal formation after vascular injury [31,32]. According to Ferns et al., expression of PDGF and PDGF receptors was upregulated upon vessel injury, and a polyclonal antibody to PDGF showed an inhibitory effect on neointimal hyperplasia in balloon-injured artery [32]. PDGF receptor tyrosine kinase inhibitors such as imatinib also showed an inhibitory effect on neointimal hyperplasia [33,34]. Sunitinib is a multi-target inhibitor of receptor tyrosine kinases including vascular endothelial growth factor (VEGF) receptor and PDGF receptor subtypes, so it could be a potential drug candidate for inhibition of neointimal hyperplasia [35,36]. Recently, Ishii et al. reported that orally administered sunitinib significantly inhibited neointimal hyperplasia in balloon-injured rat carotid artery by reducing cell proliferation [36]. Sanders et al. introduced a PLGA perivascular bilayer wrap device for which sunitinib was used as a model drug [35]. They confirmed that high amounts of the drug remained in the vascular segment for up to four weeks using a porcine model [35]. In our study, sunitinib also showed a similar inhibitory effect on neointimal formation to the other tested drugs. Similar to sirolimus-treated group, the PCNA-positive cell level of the sunitinib group was lower than I + D group. In addition, apoptotic ratio was lower than that of the paclitaxel groups and similar to that of the MN device-only group. For these reasons, local delivery of sunitinib appears promising for prevention of neointimal hyperplasia in various vascular obstructive diseases. In particular, since VEGF is also known to be involved in neointimal formation at the surgical anastomosis site of the vessel [35], sunitinib is expected to be more effective for prevention of restenosis after vascular anastomosis, such as coronary artery bypass grafting.

The relatively high ratio of apoptotic cells observed in the I + D group is another important discussion point. According to the study of Roque et al., the ratio of apoptotic cells increased until the first week after balloon injury [37]. However, apoptotic cells gradually decreased and were rarely seen in the vessel by the fourth week [37]. The first possible reason for this discrepancy is the possibility of overestimation of apoptotic cells in the present study. Generally, it is accepted that TUNEL positivity is not synonymous with apoptosis [38]. The TUNEL assay can stain pre-apoptotic cells that may not proceed to apoptosis and do not have apoptotic morphology [38]. The staining pattern and nuclear morphology in other groups was quite different from the apoptotic nuclei in the paclitaxel group, which were believed to be certain apoptotic cells. Therefore, it is possible that TUNEL-positive results in other groups might have included pre-apoptotic cells. In particular, the finding of few differences in wall thickness and structures between the device-introduced artery and a normal artery could be evidence for this hypothesis. As another possible reason, the relatively rigid base of the device and outer Tygon tube were likely to have negative effects on the vessel. Continuous shear stress to the blood vessel wall has been reported to induce arterial dilatation [39]. However, whether mechanical stress from the device is directly related to cell apoptosis cannot be determined with this study design. Further comparison study using a flexible microneedle device is required.

In conclusion, this study demonstrated effective drug delivery by a perivascular MN device. Inhibitory effects of neointimal formation by paclitaxel, sirolimus, and sunitinib were also identified. However, considering its capability for slow drug release, sirolimus and sunitinib appear to be more appropriate drugs for this device because they showed effective suppression of neointima formation with lower effect on the vascular wall thickness. An assessment of long-term safety study is planned for a follow-up study.

## Figures and Tables

**Table 1:** Summary of quantitative analysis results. The measured neointimal area and % of neointimal formation were significantly decreased in all drug-treated groups. However, the tunica media layer of the paclitaxel-treated group was significantly thinner than that of other groups and also showed the highest apoptotic ratio (I + D: balloon injury + MN device only group; I + Sir: balloon injury + sirolimus-loaded group; I + Sun: balloon injury + sunitinib-loaded group; balloon injury + paclitaxel-loaded group; -: not assessed; *** *p* < 0.001 when compared with other groups; ** *p* < 0.01 when compared with I + Ptx group; * *p* < 0.05 when compared with I + D group; ^††^ *p* < 0.01 when compared with I + Sir group and I + Sun group).

Parameters	Normal artery	I + D	I + Sir	I + Sun	I + Ptx
Neointimal area (mm^2^)	-	0.56 ± 0.13 ***	0.14 ± 0.08	0.15 ± 0.04	0.26 ± 0.10
Tunica media area (mm^2^)	-	1.09 ± 0.18	1.24 ± 0.31	1.23 ± 0.37	0.82 ± 0.23
Luminal area (mm^2^)	1.67 ± 0.31	1.05 ± 0.64	1.94 ± 0.59	1.42 ± 0.47	4.90 ± 0.90 ***
Neointimal formation (%)	-	33.59 ± 19.85 ***	7.42 ± 5.59	9.02 ± 4.08	4.70 ± 2.31
Neointimal area/tunica media area (%)	-	52.57 ± 13.92 ***	11.26 ± 6.80 **	12.86 ± 4.07 **	33.43 ± 11.62 *
Tunica media thickness (mm)	0.20 ± 0.05	0.18 ± 0.04	0.19 ± 0.05	0.17 ± 0.04	0.07 ± 0.02 ***
PCNA-positive cells (%)	3.03 ± 1.37	70.82 ± 5.11 ***	33.59 ± 7.26	32.59 ± 2.49	14.82 ± 0.46 ^††^
TUNEL-positive cells (%)	6.58 ± 2.27 ***	38.33 ± 3.30	37.99 ± 8.78	34.71 ± 4.48	85.60 ± 7.14 ***
